# Tetrodotoxins in Ribbon Worms *Cephalothrix* cf. *simula* and *Kulikovia alborostrata* from Peter the Great Bay, Sea of Japan

**DOI:** 10.3390/toxins15010016

**Published:** 2022-12-27

**Authors:** Anna E. Vlasenko, Timur Yu. Magarlamov

**Affiliations:** A.V. Zhirmunsky National Scientific Center of Marine Biology, Far Eastern Branch, Russian Academy of Sciences, 690041 Vladivostok, Russia

**Keywords:** tetrodotoxin, tetrodotoxin analogues, TTX, HPLC–MS/MS, Nemertea

## Abstract

Tetrodotoxin, an extremely potent low-molecular-weight neurotoxin, and its analogues (TTXs) are widely distributed in aquatic and terrestrial ecosystems. Most investigations concerning TTXs have been conducted mainly on puffer fish, octopus, and mollusks, without paying due attention to various non-edible animals including nemerteans, a small group of marine worms, several species of which have been shown to possess high amounts of TTXs. In this study, for the first time, variations in TTX and its analogues, in 32 specimens of *Cephalothrix* cf. *simula* and 36 specimens of *Kulikovia alborostrata*, from Peter the Great Bay Sea of Japan were investigated, which may contribute to elucidation of TTXs migration pathways in ecosystems. Using high performance liquid chromatography with tandem mass spectrometry (HPLC–MS/MS), it was found that the total TTXs concentrations within both species vary by one to several orders of magnitude, 85.75–7108.26 µg/g and 0.35–8.11 ng/g in *C*. cf. *simula* and *K. alborostrata*, respectively. The intra- and interspecies similarities in proportions of TTXs in both species were observed; based on the results, a possible way of their toxification was discussed.

## 1. Introduction

Tetrodotoxin (TTX) is an extremely potent low-molecular-weight sodium channel blocker that originates from bacteria and is widespread in aquatic and terrestrial ecosystems. It is responsible for seafood poisoning events in the countries of the Indo-Pacific region [1], where it was recorded to cause paralysis and respiratory and/or heart failure, in severe cases. Recently, invasive TTX-bearing species have become increasingly widely distributed in waters of Europe, North and South America, and Oceania, thereby expanding the geography of TTX poisoning [2]. It has also been reported that in animals, TTX usually co-occurs with its analogues [3], of which several are more potent than TTX itself [4].

Nemertea is a phylum of marine worms, also known as ribbon worms, comprising more than 1350 species [5], of which most are active predators. Among nemerteans, TTX was first found in 1988 in *Lineus fuscoviridis* and *Tubulanus punctatus* [6]. Subsequently, a number of TTX-bearing nemerteans were identified from all three classes of Nemertean (Palaeonemertea, Pilidiophora, and Hoplonemertea); those included extremely toxic species whose TTXs level reaches those recorded from pufferfish, octopus, and newts [7,8,9,10]. Currently, TTX profiles of different organisms attract the increasing attention of researchers aiming to elucidate the accumulation mechanisms and migration pathways of TTXs in ecosystems. Previously, a study of concentrations of TTX and its analogues in extremely toxic *C.* cf. *simula* specimens from different habitats revealed a wide variation in levels of toxins. Thus, using a mouse bioassay, which is a less precise method than high performance liquid chromatography with tandem mass spectrometry (HPLC–MS/MS), Asakawa et al. [10] estimated the toxicity of the *C. simula* population that inhabits Hiroshima Bay (Japan) to be equivalent to 30.08–4555.02 µg/g of TTX per 1 g of body weight. Nevertheless, they did not provide information about concentrations of the TTX analogues separately. Another study of *C. simula* collected at Godrevy Point, Cornwall, England, described the proportions of separate TTX analogues without evaluating the range of TTXs concentrations, since TTXs were quantified in only a single specimen [11]. An earlier published study with TTX quantification in a pooled sample from seven specimens of *K. alborostrata*, showed that it contained < 0.6 ng/g [12]. In another study, only qualitative TTXs detection was carried out. 

In the present report, the concentrations of TTX and its analogues in two species of nemerteans, the only TTX-containing animals known from the Russian coast of the Sea of Japan were assessed. TTXs profiles for 32 specimens of *C.* cf. *simula* and 36 specimens of *K. alborostrata*, collected from Spokoynaya Bay, Peter the Great Bay, were analyzed, and based on the results, the possible way of their toxification was suggested. 

## 2. Results

**Figure 1:**
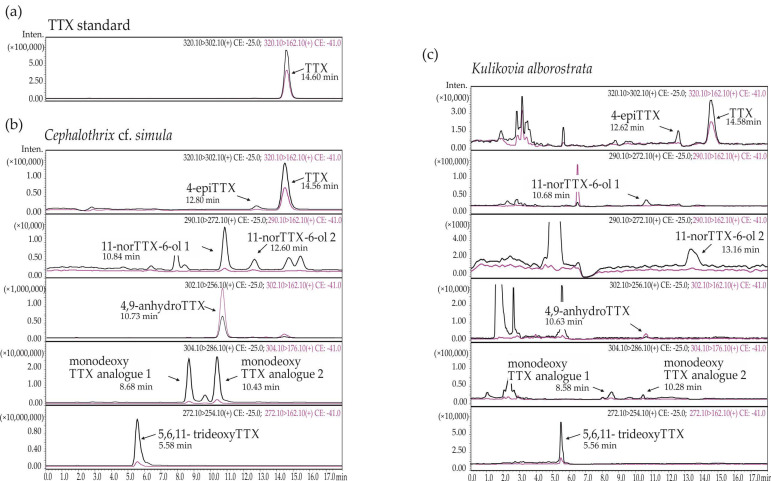
Representative high-performance liquid chromatography–tandem mass spectrometry (HPLC–MS/MS) chromatograms of (**a**) tetrodotoxin (TTX) standard and (**b**)TTX and its analogues from *Cephalothrix* cf. *simula*, and (**c**) *Kulikovia alborostrata*. The black and red lines represent two different mass transitions (described in each chromatogram).

**Figure 2:**
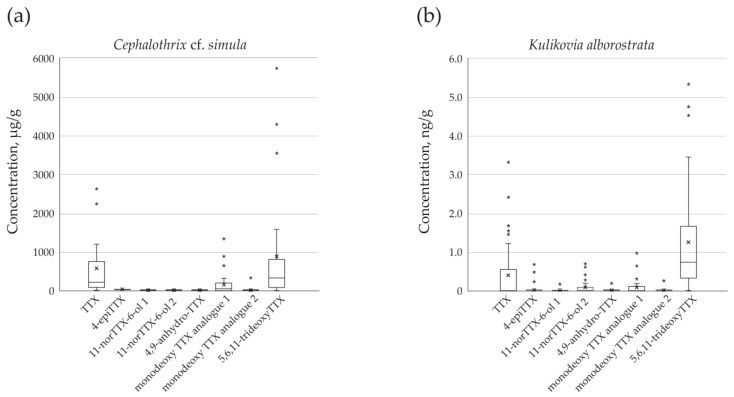
Box and whisker plot representing the mean and median TTXs concentrations of (**a**) *Cephalothrix* cf. *simula* (*n* = 32) and (**b**) *Kulikovia alborostrata* (*n* = 36). Lower and upper box boundaries represent 25th and 75th percentiles, respectively; lines inside represents box median; lower and upper error lines reflect the variability outside these percentiles; asterisks (*) indicate outlying values; and an x mark indicates the mean value.

Eight TTXs were detected in extracts of *C.* cf. *simula* (Table 1, Figure 1a, b), with their total concentration varying from 85.75 to 7,108.26 µg/g of nemertean body weight. The toxins TTX and 5,6,11-trideoxyTTX showed the highest representation: their mean values were 601.91 ± 774.28 (from 27.11 to 2677.51 µg/g) and 886.52 ± 1394.02 µg/g (from 13.35 to 5737.72 µg/g), respectively; the third major toxin was monodeoxy TTX analogue 1, that amounted to 157.47 ± 291.09 µg/g (from 3.03 to 1340.96 µg/g) (Figure 2a).

The extracts of *K. alborostrata* contained from one to six TTXs (Table 2, Figure 1a, c), with a total concentration of 0.35–8.11 ng/g of nemertean body weight. The toxin with the highest representation was 5,6,11-trideoxyTTX (1.28 ± 1.38 ng/g, from the level below limit of detection (LOD) to 5.33 ng/g), followed by TTX (0.42 ± 0.78 ng/g, from the level below LOD to 3.32 ng/g), monodeoxy TTX analogue 1 (0.1 ± 0.2 % ng/g, from the level below LOD to 0.98 ng/g), and 11-norTTX-6-ol 2 (0.09 ± 0.18 ng/g, from the level below LOD to 0.71 ng/g) (Figure 2b).

## 3. Discussion

According to the results of HPLC–MS/MS, the total concentration of TTXs in *C.* cf. *simula*, having a mean value of 1685.17 ± 1938.80 µg/g of nemertean body weight, varies by several orders of magnitude, from 85.75 to 7108.26 µg/g (Table 1), which fits within the range of toxin concentrations reported for this species [10,11]. The TTX concentration in the extracts of *K. alborostrata* is 1.96 ± 2.18 ng/g (with a range from 0.35 to 8.11 ng/g), i.e., significantly lower than that in the extracts of *C.* cf. *simula* (Table 1 and Table 2). Therefore, the level of toxins in both *C.* cf. *simula* and *K. alborostrata*, within and between separate populations, can vary by several orders of magnitude. The high difference in the toxin concentration within the same species is typical for most TTX-bearing animals, like pufferfish, mollusks, newts, etc. [13,14,15,16,17,18,19].

The toxins TTX, monodeoxy TTX analogue 1, and 5,6,11-trideoxy-TTX in the *C.* cf. *simula* individuals, analyzed in the present study, have the highest percentages, from 11.78 to 78.08% for TTX (from 27.11 to 2677.51 µg/g), from 0.25 to 56.85% for monodeoxy TTX analogue 1 (3.03 to 1340.96 µg/g), and from 14.74 to 80.73% for 5,6,11-trideoxy-TTX (from 13.35 to 5737.72 µg/g). Despite the wide variation in toxin concentrations between individuals, the total proportion of the three toxins in all specimens made up 96.78 ± 3.09% of the total amount of TTXs. The percentages of TTX, monodeoxy TTX analogue 1, and 5,6,11-trideoxy-TTX in the extracts of seven *C.* cf. *simula* specimens, collected from the same locality (Peter the Great Bay, Sea of Japan) in previous years, were similar [12,20]. In the study of 2018, the TTX percentage amounted to 21.85%; monodeoxy TTX analogue 1, 32.33%; and 5,6,11-trideoxy-TTX, 39.93% (with the total proportion of 94.11%) [12]. In 2020, the TTX percentage amounted to 33.63 ± 15.48%; monodeoxy TTX analogue 1, 17.37 ± 15.86%; and 5,6,11-trideoxy-TTX, 44.97 ± 14.72% (with the total of 97.36 ± 1.75%) [20]. A different pattern of TTX analogues was recorded from a *C. simula* caught in England: the content of TTX was 64%, followed by 6,11-dideoxyTTX (21%), the total proportion of 5-deoxyTTX (11-deoxyTTX) and 5,6,11-trideoxyTTX was 9.9% [11], and 11-oxoTTX amounted to 5%. Since only one specimen was investigated, it was impossible to draw any conclusions regarding toxin profiles of different *C.* cf. *simula* populations. Nevertheless, several studies carried out on pufferfish *Lagocephalus sceleratus*, collected off North Lebanon [21] and along the Greece coast [22], as well as *Arothron nigropunctatus* from two different localities, Okinawa, Japan, and the Solomon Islands [23] have shown that neither qualitative nor quantitative TTX compositions can be considered stable characteristics of the species. 

TTXs have an exogenous origin in TTX-bearing animals, that accumulate directly from marine bacteria, the primary TTX producers, and/or through the food web [24,25]. Since no biotransformation of TTX and its non-equilibrium analogues has been observed in living organisms [26], the intraspecies difference in qualitative and quantitative TTX profile, between specimens from different localities, may indicate its correlation with the source of toxins, which is supposed to be unique in each region. The characteristic toxin profile of each locality can originate from spectra of free-living bacteria and the microbiome of animals inhabiting it. The final TTX profile of consumers, including nemerteans, is presumably determined by their microbiome, or diet preferences (the TTX profiles of their prey items), or both. To date, there have been no studies considering the correlation between microbiome and TTX profile. However, the observations on the microbiomes of *C. simula* from England and *C.* cf. *simula* from the Sea of Japan have revealed differences in the most representative bacteria, even on the phylum level.

**Figure 3:**
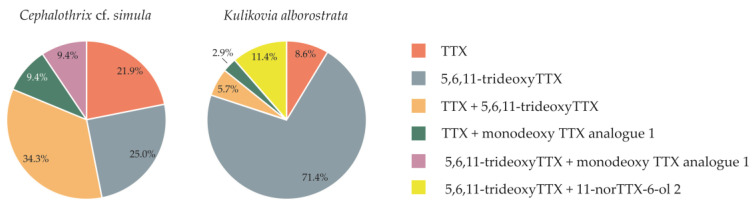
The dominant toxins in 32 specimens of *Cephalothrix* cf. *simula* and 36 specimens of *Kulikovia alborostrata*.

An interspecies comparison of the TTX profiles of *C.* cf. *simula* and *K. alborostrata* have shown that some of the major toxins were the same. All the nemertean individuals were divided into six groups, based on the toxins which comprised 60% of all toxins recorded from them: (1) TTX; (2) 5,6,11-trideoxyTTX; (3) TTX + 5,6,11-trideoxyTTX; (4) TTX + monodeoxy TTX analogue 1; (5) 5,6,11-trideoxyTTX + monodeoxy TTX analogue 1; and (6) 5,6,11-trideoxyTTX + 11-norTTX-6-ol 2. Four groups were common for *C.* cf. *simula* and *K. alborostrata*, while both species were represented by five groups each (Figure 3). The comparable compositions of the major toxins may result from similar accumulation pathways of TTX and its analogues for the two species, including obtainment from their own microbiomes and/or preferred diet. Recently, it has been reported that the microbiomes of *C.* cf. *simula* and *K. alborostrata* differ significantly [27] and, although the role of microbiome cannot be ruled out, it is probable that the levels of TTXs within both species may also include contributions from consumed prey. The effect of prey toxins profiles on predator ones has already been demonstrated by several researchers. Thus, Ito et al. [28] have shown that the TTX / 5,6,11-trideoxyTTX ratio in pufferfish (*Chelonodon patoca*) and toxic goby (*Yongeichthys criniger*) can result from this ratio in their presumable prey, the flatworm *Planocera multitentaculata.* In another study on *Octopus vulgaris* fed shellfish, containing another group of guanidinium toxins, paralytic shellfish toxins (PSTs), the similar PSTs prevailed as a result [29]. Since in most individuals of *C.* cf. *simula* and *K. alborostrata* from the same locality the major toxins are similar, TTX and 5,6,11-trideoxyTTX (Table 1 and Table 2), which presumably suggests that their toxification have common sources. This suggestion is supported by data from articles describing similar nemerteans’ dietary preferences as predators. Thus, members of the family Lineidae (which includes *K. alborostrata*) prefer mostly polychaetes, from several families (Nereidae, Phyllodocidae, Polynoidae, and Terebellidae) [30,31,32,33,34,35]. Prey preferences of the *Cephalothrix* species are poorly known, but several feeding experiments have revealed their diverse diet that includes a wide range of taxonomic classes of prey: polychaetes [36], oligochaetes, nematodes [33], and crustaceans (amphipods and isopods) [37]. Therefore, it can be assumed that several prey items, common for both nemertean species, were the sources of the same toxins in them. Nevertheless, this assumption should be further verified through dietary investigations, using DNA metabarcoding and determination of toxin profiles of the presumed prey. Additionally, the contribution of the microbiomes of *C.* cf. *simula* and *K. alborostrata* to their toxification should not be ignored, and remains an important issue to address.

## 4. Conclusions

In the present study, a wide variation in TTXs concentrations have been observed in extracts of *C.* cf. *simula* and *K. alborostrata*. The overlaps of the TTXs compositions of the two species may indicate that both accumulate (at least some part of) toxins from several common sources, including their own microbiomes and/or preferred diet. The obtained TTXs profiles and those reported in the literature have been compared; and as a result, the assumption has been made that TTXs profiles are specific for each region. Further investigations of toxin profiles of different organisms are expected to elucidate the migration pathways of TTXs and its analogues in ecosystems.

## 5. Materials and Methods

### 5.1. Sample Collection

**Figure 4:**
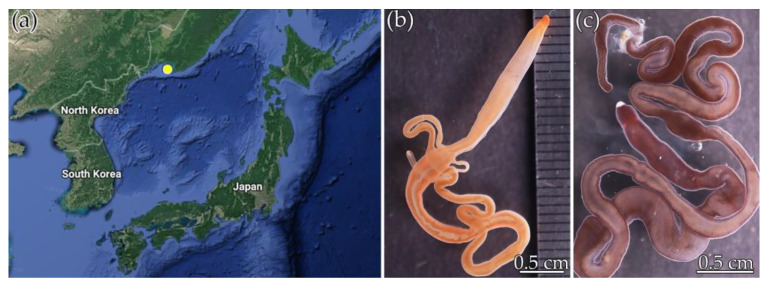
Sampling location (**a**) of *Cephalothrix* cf. *simula*, and (**b**) *Kulikovia alborostrata.* (**c**) The images of nemerteans were taken with a reflex camera in a macro mode.

*Cephalothrix* cf. *simula* (32 specimens) and *Kulikovia alborostrata* (36 specimens) were obtained from rhizoids of the biennial brown alga *Saccharina* sp., collected at a depth of 0.5–1.5 m in Spokoynaya Bay, Peter the Great Bay, Sea of Japan (42.7090° N, 133.1809° E), in May–August 2020 (Figure 4). After collection, the rhizoids were moved to the Vostok Marine Biological Station of the A.V. Zhirmunsky National Scientific Center of Marine Biology, Far Eastern Branch, Russian Academy of Sciences (Vladivostok, Russia) and placed in tanks with seawater at 20 °C, and kept there until nemerteans came out of them. The nemertean species were identified based on morphological characters, by Dr. Alexey V. Chernyshev, an expert in nemertean biology from the A.V. Zhirmunsky National Scientific Center of Marine Biology. Before extraction, the animals were kept in tanks, with aerated seawater at 17 °C. 

### 5.2. Materials

All chemicals used were of analytical grade and were used as received, without any further purification and were supplied by Sigma-Aldrich, St. Louis, MO, USA. TTX solution was supplied by Alomone Labs Ltd., Jerusalem, Israel.

### 5.3. Extraction of TTX and its analogues 

The nemertean extracts were prepared by the following procedure. The samples were homogenized in a 0.1% solution of acetic acid in 70% methanol (the sample/solution ratio was 1:10 *v*/*v*) for 5 min, using a hand-held homogenizer, and then ultrasonicated using a Sonopuls HD 2070 homogenizer (Bandelin, Berlin, Germany) for 10 min (at a frequency of 20 kHz; amplitude, 228 µm; working cycle, 0.8 s; and interval, 0.2 s). The homogenates were centrifuged (14,000 × *g*, 10 min, 4 °C), and the supernatants were collected. The remaining precipitates were extracted twice more, in a 0.1% solution of acetic acid in 70% methanol (the sample/solution ratio was 1:2 *v*/*v*), and the supernatants were pooled. The extracts were evaporated in a rotary evaporator (Labconco, Kansas City, MO, USA) at 60 °C. The dry precipitates were dissolved in a 0.1% aqueous solution of acetic acid, at 1 mL/g of nemertean tissue, and concentrated by ultrafiltration on a Vivaspin turbo concentrator (nominal cutoff molecular weight of 5 kDa (Sartorius, Goettingen, Germany)). The resulting samples were stored at –20 °C for further analysis.

### 5.4. Analysis of TTX and its analogues by HPLC–MS/MS

TTX and its analogues were identified by HPLC–MS/MS. The HPLC system included two pairs of LC-30 pumps, a SIL-30AC autosampler, a CTO-20A thermostat, an SCL-20A system controller, and a triple quadrupole mass spectrometer LCMS-8060 (ShimadzuEuropa, Duisburg, Germany), with electrostatic spray ionization (ESI). Separation was carried out on a SeQuant ZIC HILIC column (150 × 2.1 mm, 5 μm) (Merck, Darmstadt, Germany) at 40 °C and a flow rate of 0.2 mL/min. A binary gradient was used: mobile phase A, ammonia (5 mM) and formic acid (8 mM) in 94:6 acetonitrile/water; and mobile phase B, ammonia (10 mM) and formic acid (20 mM) in water. A gradient profile was used as follows: (a) 0–4.3 min, 15% B; (b) 4.3–16 min, 25% B; and (c) 16–20 min, 50% B. The sample volume was 1 μL. A SeQuant ZIC-HILIC guard column (20 × 2.1 mm, 5 μm) (Merck, Darmstadt, Germany) was installed in line, before the analytical column through a two-position 6-port valve. At 4.4 min, the valve was switched, and the guard columns were backflashed with isopropanol (4.4–9 min) and water (9–15 min), at a flow rate of 0.3 mL/min. At 16 min, the valve was switched back. The mass spectrometer was operated in the scan (m/z 200–1,000) at multiple reaction monitoring (MRM) modes. The ion source parameters were as follows: interface temperature, 380 °C; desolvation line temperature, 250 °C; nebulizing gas (N_2_) flow, 3 L/min; drying gas (N_2_) flow, 3 L/min; and heating gas (dry air) flow, 17 L/min. Collision energy was 41 eV for precursor transition and 25 eV for fragment transitions. The TTX concentration was calculated using the calibration curve of a standard TTX solution series. The toxins detection criteria included a precursor MRM transition peak S/N ratio > 3, and a relative intensity of the fragment ion peak > 4%. TTX analogues were detected without using the standards, according the order of toxins elution, similar to that described by Bane et al. [4], where the same SeQuant ZIC-HILIC guard column was used. The concentrations of TTX analogues were calculated following the procedure of Chen et al. [38], using the TTX standard as a reference peak. The method was validated using standard TTX solutions in the MRM mode. The linearity range was from 0.6 to 100 ng/mL; the recovery range from 1 to 100 ng/mL of TTX was 98.4%; the limit of quantification was determined as S/N = 10 and was 0.6 ng/mL; the LOD was determined as S/N=3 and was 0.2 ng/mL; and the relative SD was 4.5–14.6%.

## Figures and Tables

**Table 1:** Concentrations of tetrodotoxin (TTX) and its analogues in extracts of *Cephalothrix* cf. *simula*.

Sample no.	Date of Collection	Toxins, µg/g								
		TTX	4-epi TTX	11-norTTX-6-ol 1	11-norTTX-6-ol 2	4,9-Anhydro-TTX	Monodeoxy TTX Analogue 1	Monodeoxy TTX Analogue 2	5,6,11-Trideoxy-TTX	Total
1	17 May 2020	764.88	31.45	0.37	2.08	2.84	56.91	1.83	3538.99	4399.33
2	16 June 2020	91.85	9.85	0.10	4.23	0.67	61.79	0.99	301.22	470.70
3	16 June 2020	524.84	21.09	0.61	0.60	4.51	306.33	0.62	3596.88	4455.47
4	3 July 2020	2630.35	19.18	0.03	0.04	13.72	21.48	-	762.12	3446.91
5	3 July 2020	98.91	1.26	0.06	0.03	0.37	5.22	0.41	121.86	228.11
6	3 July 2020	2274.31	34.16	5.20	-	6.10	106.23	346.29	4286.28	7058.56
7	4 July 2020	124.22	2.16	0.10	0.06	0.67	9.19	1.27	556.37	694.04
8	4 July 2020	84.17	7.01	0.04	4.81	0.32	46.44	0.56	281.13	424.48
9	5 July 2020	108.61	13.16	0.07	0.03	0.97	39.62	0.76	44.49	207.71
10	5 July 2020	108.52	9.50	0.01	0.02	1.04	29.23	1.33	106.86	256.50
11	5 July 2020	791.36	5.25	0.59	0.21	2.00	16.42	1.82	646.26	1463.93
12	7 July 2020	2677.51	16.17	-	-	4.44	9.01	33.38	860.82	3601.32
13	7 July 2020	185.25	5.84	-	-	0.81	34.56	86.94	230.07	543.46
14	7 July 2020	279.79	11.47	0.09	0.39	2.24	5.45	6.51	52.91	358.85
15	13 July 2020	2251.33	14.12	0.14	0.84	3.81	59.51	16.97	820.70	3167.42
16	13 July 2020	27.11	2.51	0.06	0.02	0.37	41.44	0.90	13.35	85.75
17	13 July 2020	78.89	5.69	0.04	0.02	1.89	261.44	0.44	111.44	459.85
18	13 July 2020	86.81	3.34	0.19	0.23	0.58	5.50	2.29	48.53	147.47
19	14 July 2020	189.84	16.68	0.03	0.02	3.49	319.14	0.82	149.94	679.95
20	14 July 2020	116.11	6.98	0.06	3.35	2.20	210.25	2.40	232.08	573.42
21	17 July 2020	223.41	30.10	0.22	0.39	4.61	891.39	2.49	540.23	1692.85
22	4 August 2020	915.69	35.86	0.42	0.12	7.29	1340.96	0.97	810.76	3112.08
23	4 August 2020	1218.41	36.05	0.28	0.17	5.48	68.51	41.63	5737.72	7108.26
24	4 August 2020	545.00	18.68	0.32	0.08	5.46	41.94	4.85	554.20	1170.53
25	4 August 2020	851.71	18.66	-	-	3.44	636.93	86.39	1195.14	2792.28
26	4 August 2020	114.15	1.13	-	-	0.18	3.03	9.09	54.53	182.11
27	4 August 2020	112.96	1.02	0.45	-	-	5.43	2.28	56.42	178.55
28	4 August 2020	532.84	1.61	-	-	-	3.84	16.24	127.90	682.43
29	4 August 2020	381.59	2.36	0.18	15.96	0.82	6.16	2.33	436.23	845.63
30	4 August 2020	204.75	5.31	0.19	4.64	5.27	18.27	0.26	124.42	363.09
31	4 August 2020	271.18	14.44	0.05	0.24	1.36	138.16	27.34	1582.99	2035.76
32	4 August 2020	394.95	12.24	0.12	0.11	5.77	239.16	0.61	385.70	1038.66

-: not detected.

**Table 2:** Concentrations of tetrodotoxin (TTX) and its analogues in extracts of *Kulikovia alborostrata*.

Sample no.		Toxins, ng/g								
	Date of Collection	TTX	4-epiTTX	11-norTTX-6-ol 1	11-norTTX-6-ol 2	4,9-anhydroTTX	Monodeoxy TTX Analogue 1	Monodeoxy TTX Analogue 2	5,6,11-trideoxyTTX	Total
1	16 May 2020	2.42	0.54	-	0.30	-	0.65	-	3.03	6.94
2	17 May 2020	3.32	0.25	-	-	0.2	0.40	0.27	2.35	6.79
3	18 May 2020	1.57	0.49	-	0.35	-	0.37	-	5.33	8.11
4	19 May 2020	-	-	-	0.71	-	-	-	2.30	3.01
5	20 May 2020	1.69	0.69	-	0.61	-	0.98	-	-	3.97
6	12 June 2020	0.21	-	-	-	-	0.07	-	0.91	1.19
7	12 June 2020	-	-	-	-	-	-	-	0.71	0.71
8	12 June 2020	-	-	-	-	-	-	-	0.63	0.63
9	12 June 2020	-	-	-	-	-	-	-	1.71	1.71
10	12 June 2020	-	-	0.18	-	-	0.11	-	1.58	1.87
11	12 June 2020	-	-	-	-	-	-	-	0.73	0.73
12	12 June 2020	-	-	-	-	-	-	-	0.35	0.35
13	12 June 2020	-	-	-	-	-	-	-	1.04	1.04
14	12 June 2020	-	-	-	0.42	-	0.32	-	0.45	1.19
15	12 June 2020	-	-	-	0.21	-	0.12	-	0.21	0.54
16	16 June 2020	1.23	0.25	-	0.15	-	-	-	3.47	5.10
17	16 June 2020	-	-	-	0.13	-	0.19	-	0.37	0.69
18	16 June 2020	-	-	-	-	-	-	-	0.45	0.45
19	16 June 2020	0.22	-	-	0.28	-	-	-	0.26	0.76
20	16 June 2020	-	-	-	-	-	0.11	-	0.38	0.49
21	16 June 2020	-	-	-	-	-	0.11	-	0.25	0.36
22	16 June 2020	0.63	-	-	-	-	-	-	4.77	5.40
23	16 June 2020	0.21	-	-	-	-	-	-	1.98	2.19
24	16 June 2020	-	-	-	-	-	-	-	0.74	0.74
25	16 June 2020	1.48	-	-	-	-	-	-	4.55	6.03
26	16 June 2020	0.22	-	-	-	-	-	-	0.32	0.54
27	16 June 2020	0.62	-	-	-	-	-	-	-	0.62
28	16 June 2020	-	-	-	-	-	-	-	1.35	1.35
29	16 June 2020	-	-	-	-	-	-	-	0.74	0.74
30	16 June 2020	0.64	-	-	-	-	-	-	-	0.64
31	16 June 2020	-	-	-	-	-	-	-	0.76	0.76
32	16 June 2020	0.47	-	-	-	-	-	-	0.27	0.74
33	16 June 2020	0.23	-	-	-	-	-	-	0.88	1.11
34	16 June 2020	-	-	-	-	-	-	-	1.16	1.16
35	16 June 2020	-	-	-	-	-	-	-	1.46	1.46
36	16 June 2020	-	-	-	-	-	-	-	0.42	0.42

-: not detected.

## Data Availability

Not applicable.
